# Inter-Individual Responses to a Blueberry Intervention across Multiple Endpoints

**DOI:** 10.3390/nu16060895

**Published:** 2024-03-20

**Authors:** Yueyue Wang, Crystal Haskell-Ramsay, Jose Lara Gallegos, John K. Lodge

**Affiliations:** 1 Department of Applied Sciences, Faculty of Health and Life Sciences, Northumbria University, Newcastle-upon-Tyne NE1 8ST, UK; yueyue.wang@northumbria.ac.uk (Y.W.); jose.lara@northumbria.ac.uk (J.L.G.); 2 Department of Psychology, Faculty of Health and Life Sciences, Northumbria University, Newcastle-upon-Tyne NE1 8ST, UK; crystal.haskell-ramsay@northumbria.ac.uk; 3 Nutrition Trials at Northumbria (NUTRAN), Northumbria University, Newcastle upon Tyne NE1 8ST, UK; 4 School of Human Sciences, London Metropolitan University, London N7 8DB, UK

**Keywords:** dietary intervention, blueberry, vascular function, cognition, individual response, inter-individual variation

## Abstract

Inter-individual variation exists in response to diet and in the endpoints related to vascular diseases and cognitive impairment. Therefore, the evaluation and characterisation of responses to a dietary intervention targeting these endpoints is important. A dietary intervention with 37 participants has been performed comparing two forms of blueberry, either whole fresh blueberry (160 g), freeze-dried blueberry powder (20 g) or a placebo control (microcrystalline cellulose), in a 1-week single-blinded cross-over randomised controlled trial (RCT) in a healthy population. The response to the intervention was calculated for each endpoint using the percentage change (±%) compared to the baseline. Extensive inter-individual variation was found in vascular health parameters (−141 to +525%) and cognitive domains (−114 to +96%) post-intervention, but there was no consistent response following the two interventions between and within participants for each endpoint measured. No significant putative discriminating urinary metabolites between interventions were found using supervised multivariate analysis. Although several discriminatory metabolites were found between the responder and non-responder groups, it was not possible to identify predictors of the response using receiver operating curve analysis. To conclude, this is the first blueberry intervention applying quartile divisions to characterise individual responses in vascular and cognitive endpoints following a specific dietary intervention; however, we did not find any consistency in the individual responses to the interventions, and we could not identify a predictive urinary metabolite as a potential biomarker for differentiation between responders and non-responders. However, the overall approach of defining a metabolic signature of response could be used in the future for tailored personalised nutritional advice.

## 1. Introduction

A healthy diet is at the forefront of the factors that are known to reduce the risk of degenerative disease. Most nutritional guidelines are based on the population as a whole, yet this does not take into account a proportion of the population that respond differently and therefore have increased or decreased requirements. It is well known that there is large inter-individual variation in the response to diet [1,2], and understanding the inter-individual variation in the response to dietary interventions is important in understanding the efficacy in improving health at the individual level. Factors that influence inter-individual variation include differences in the absorption, metabolism, tissue distribution, bioavailability [3] and functionality of nutrients [1,2]. Nutrigenetic approaches investigating single-nucleotide polymorphisms can identify sources of variation as polymorphisms in genes influencing the aforementioned processes that will impact the availability, function and ultimately clinical endpoints and risk of disease [4]. For instance, after ingestion in the gastrointestinal tract, the enzyme catechol-O-methyltransferases (COMT, AA or GG genotype), at the genetic level, is involved in anthocyanin metabolism and could play a role in inducing variability in the response to an intervention [5]. The gut microbiome also plays an important role in the inter-individual variation in response to diet through the effect on bioactive compounds [1], and its diversity may help to explain the differential metabolism of a variety of plant bioactives [6] and how this then has an effect on a biological system such as blood pressure or cognition. Furthermore, the functionality of the microbiome towards food components can also influence variation, as the microbiome has the ability to modify the nutritional status through the conversion of food components into active species [7,8].

We recently reviewed and meta-analysed the role of fruit in cardio-protection and cognition, which highlighted variations in the response to fruit within a number of clinical endpoints [9,10]. For example, from 21 dietary interventions using fruit or fruit juices, the response for systolic blood pressure (BP) ranged between a 4% increase and a 9% decrease; for total cholesterol, the response ranged between a 15% increase and a 13% decrease; and, similarly, for outcomes related to cognition, there was a 9% increase to a 23% decrease in memory, and a 10% increase and to 4% decrease in executive function. These data highlight that the variation in response to a fruit intervention across biological endpoints is extensive. However, there is a lack of evidence to identify whether individual’s response is consistent across biological endpoints or whether the response is inconsistent. For example, if a certain participant responds favourably in terms of blood pressure, do they also respond similarly to an endpoint related to blood pressure (e.g., plasma nitrite) or in a different biological system, e.g., cognitive endpoints? A consistent response will help to identify a sustainable approach to improve health and prevent disease. Metabolomics is proving to be a useful tool in nutritional research and has been used previously to investigate variation in response. For example, Garcia-Perez et al. developed a prospective dietary metabotype score to directly map individual urinary metabolites to differentiate metabotypes [11], and this score was used to classify individual responses to either a healthy or unhealthy diet based on World Health Organization (WHO) guidelines [11], and, when the metabotypes were profiled, the score could be used to predict the diet that they were adhering to.

Generally, blueberries are described as a “super fruit” and are popular among consumers [12]. This is mainly due to their rich polyphenol content; they have shown a high antioxidant capacity in vitro, characterised by various types of phytochemicals, including abundant anthocyanin pigments [12]. A number of epidemiological studies and RCTs have linked the regular consumption of blueberries and/or anthocyanins with a reduced risk of cardiovascular disease, obesity, type 2 diabetes, neuroprotection and cognitive maintenance [13,14,15,16]. Freeze drying is one of the most commonly used methods for their preservation and consumption [12]. The aim of the present study was to characterise the consistency of the variation in multiple endpoints in response to the blueberry interventions that we have recently published [13], and to use the urinary metabolomic differences between responders (RS) and non-responders (NRS) in the intervention to identify predictors of the response.

## 2. Materials and Methods

### 2.1. Participants

The study was approved by Northumbria University’s Faculty of Health and Life Sciences Ethics Committee (reference: 10113) and was registered under ClinicalTrials.gov (ID: NCT04015258) and reported by Wang et al. (2022) [13]. Thirty-five participants were required, based on data from two previous studies targeting cardiovascular health and cognition [17,18]. An effect size of d = 4.7 and −1.83 was calculated separately and indicated that a total of 35 healthy subjects were required to detect an effect difference in treatments at a two-sided 0.05 significance level with statistical power of 0.8.

### 2.2. Treatment

There were two experimental arms, namely the whole blueberry intervention arm and freeze-dried blueberry powder intervention arm, with one placebo arm implemented as a control. Fresh blueberries (*Vaccinium corymbosum* L.) were purchased from a local grocer that used the same source (Spanish origin) throughout the intervention; freeze-dried blueberry powder was purchased from Lio-Licious freeze-dried fruits (Lio-Licious, Preston, UK); the microcrystalline cellulose control was purchased from Blackburn Distributions.

The powder to fresh blueberry net weight conversion was provided by the supplier. The systematic review and meta-analysis of RCTs suggested that at least 1 week is needed for the assessment of berry fruits overall regarding the improvement of vascular and cognitive health, specifically to significantly improve SBP and DBP [9,10]. Our review also found that interventions supplementing berries (e.g., blueberry, 100 mg extract powder, 200 g whole blueberry equivalent, 6–24 weeks) with a sufficient sample size appeared to have the greatest potential to improve cognition in both young and old adults [10]. The study supplied either 160 g of fresh whole blueberries (4 handfuls or 2 NHS adult portion sizes), which were weighed prior to each study visit and provided to the participants (the blueberries were packed in 7 separate food-grade sealed bags and participants were told to store the blueberries in a refrigerator and take one bag out for consumption daily, preferably consumed prior to lunch), or 20 g of freeze-dried blueberry powder (equivalent to 160 g of whole fresh blueberries) packed in a sachet prior to each study visit and provided to the participants. The blueberry powder was measured with a tablespoon provided with a 20 g marker line, which was validated with Benchtop Electrical food scales. Participants were told to store the blueberry powder in a cool and dry place and take one tablespoon of the powder (measured with the marker line) for consumption daily. It was suggested to mix and consume the powder with water at room temperature, preferably prior to lunch. Finally, the control capsule was a plant-derived fibre microcrystalline cellulose (participants were blinded to the fact that this contained encapsulated blueberry extract components) and the 7 capsules required for the intervention were packed in food-grade capsule containers; the participants were told to take 1 capsule with water for consumption daily, preferably prior to lunch. Participants were administered these treatments on separate occasions for 1 week, with a 1-week washout period between each. Each participant was given each treatment in a random sequence.

Participants were provided with a list of polyphenol-rich foods and sources of dietary nitrates and nitrites to avoid throughout the intervention (Supplementary Material), and they were asked to provide a food diary for the day prior to fasting for each study visit, so that the researchers could check whether they had consumed the blueberries. Participants were also required to complete a questionnaire investigating their awareness of the placebo control at the end of the trial.

Random permutation was performed by an online randomisation tool (http://randomization.com/, accessed on 1 November 2018); the blinding of the control treatment was conducted by the researcher (Y.W.) who coordinated the intervention.

### 2.3. Clinical Endpoints

For the vascular function endpoints, there were 9 assessment parameters. Systolic and diastolic blood pressure (SBP and DBP) and the pulse wave velocity of the carotid artery and radial artery (crPWV) were assessed at baseline and post-intervention. PWV was the velocity assessed by the brachial–radial distance (m) dividing by pulse wave time (s) from the carotid artery to the radial artery, and was presented in the unit of m/s. The PWV of the carotid artery and radial artery (crPWV) was assessed using a SphygmoCor (ScanMed medical, București, Romania) device, and the cardiac rhythm was monitored using an electrocardiogram (ECG) pad. The readings were repeated 3 times. PWV was measured based on a validated protocol [19]. SBP and DBP were measured 3 times by a vital signs monitor (GE Carescape), with participants sitting in an upright position for 5 min. Readings were taken at >1-min intervals. Plasma was used to measure the concentration of glucose (the Randox Daytona GOD-PAP (Cat. No. GL8038), with a measuring range of 0.200–35.5 mmol/L, was used; a correlation of r = 0.999 against another commercially available method was reported) and the lipid status (total and HDL-, LDL-cholesterol, triglycerides) (Randox Daytona GOD-PAP with a measuring range of 0.22–21.7 mmol/L (Cat. No. CH200) and 0.1–13.4 mmol/L (Cat. No. TR210) for total cholesterol and triglycerides, respectively). The Randox Daytona Direct Clearance Method was used (Cat. No. CH1383) to measure HDL-cholesterol, with a measuring range of 0.189–4.03 mmol/L. A correlation of r = 0.999 against another commercially available method was reported. The LDL-cholesterol level was further calculated using the Friedewald equation: LDL-cholesterol [mmol/L] = total cholesterol [mmol/L] − HDL-cholesterol [mmol/L] − triglyceride/5 [mmol/L] [20]. The nitric oxide (NO) metabolite nitrite (NO_2_^−^) was also measured (the total amount of nitrite [NO_2_^−^] in deproteinised plasma was determined by using the chemiluminescence method via a purge system from Sievers Instruments (model NOA 280i, Boulder, CO, USA), with repeatability of ±5%. The plasma samples used for nitrite analysis were deproteinised by using ethanol to prevent foaming. Plasma samples were mixed with ethanol in a 2-fold dilution in microcentrifuge tubes and left to stand for 30 min. The microcentrifuge tube was centrifuged at 12,500 rpm for 5 min. The supernatant was then removed for analysis. Standard solutions containing 10 nM, 50 nM, 100 nM, 1 µM, 5 µM, 10 µM, 50 µM and 100 µM of sodium nitrite [NaNO_2_] were prepared with nitrate-free deionised water and analysed to construct a calibration curve at baseline and post-intervention.

For cognition endpoints, there were 7 cognitive domains (endpoints) assessed by 43 test parameters. Domains of working memory as assessed by serial 3 s and 7 s tasks; episodic memory as assessed by immediate and delayed word recall and word recognition tasks, attention as assessed by digit vigilance and mood (calm, alert and content) and mental fatigue as assessed by a visual analogue scale were also evaluated at baseline and post-intervention. A battery of computerised cognitive tests that took approximately 30 min to complete was applied on each study day. These tasks were provided by a software program called the Computerised Mental Performance Assessment System (COMPASS Version 1.0, Northumbria University), which has shown stable sensitivity to a range of nutritional interventions [16,21]. The participants were trained on the computerised cognitive tasks on the screening visit prior to the assessment visits and were provided with noise-cancelling headphones during testing in a monitored quiet environment.

The inclusion and exclusion criteria, detailed study procedure and statistical analyses applied to summarise the treatment effects in the main study were the same as those reported by Wang et al. (2022) [13]. A retrospective post hoc power calculation was performed for the main study as multiple primary endpoints were assessed.

### 2.4. Characterising Responses to Interventions

The response for each endpoint was calculated from the change from baseline and presented as a percentage (%). For each endpoint, participants were ranked based on their response from highest to lowest. Then, participants that ranked at the upper quartile (Q1) or the lower quartile (Q4) were identified, considering the effect direction of each assessed endpoint. There were 43 test parameters measuring the 7 main cognitive endpoints and 9 vascular function endpoints; thus, there were 52 endpoints in total. Finally, based on the ranking of the number of test parameters that participants responded to from the largest to the smallest, the Q1 participants were identified as the RS group. Similarly, based on the ranking of the number of test parameters that participants non-responded to from the largest to the smallest, the Q4 participants were identified as the NRS group. Those participants belonging to the Q2 and Q3 quartiles of response were characterised as average responders. To assess the effect of gender, BMI and the visit order on the response group, a chi-square test was applied. The participants recruited in the current study were mostly young (97% of participants being less than 42 years old), so the association of age and response to clinical endpoints was not assessed.

### 2.5. Metabolite Profiling

For urine sample preparation, urine (100 µL) was mixed with the same volume of chilled LC-MS-grade methanol (−20 °C) containing 0.125% formic acid, mixed and allowed to chill in a freezer (−4 °C) for 30 min. The sample was then centrifuged for 2 min at 4 °C at 10,000 rpm and then carefully transferred to a sample vial for HILIC analysis. QCs were prepared by pooling an aliquot of all samples. Details of the LC-MS conditions can be found in a previous publication [22]; essentially, urine samples were analysed on a Dionex 3000 Ultra High-Pressure Liquid Chromatography (UHPLC) system connected to a Q-Exactive high-resolution mass spectrometer system (ThermoScientific, Bremen, Germany). Following LC-MS analysis, peak table generation and alignment were performed using the Compound Discoverer™ 2.1 small-molecule identification software (ThermoScientific, Bremen, Germany) with an alignment window of 0.25 min, mass tolerance of 5 ppm and a signal intensity threshold of 200,000 counts, with a signal to noise ratio of 5:1. Positive mode data only were collected.

### 2.6. Characterisation and Identification of Discriminating Features

Post-data-acquisition processing and alignment was performed using Compound Discoverer™ and data were categorised into different treatment groups for analysis. Pooled QC samples and sample blanks were also included and grouped accordingly in order to assess and evaluate system stability and track potential carryover effects throughout the entire batch analysis. The resulting peak table was sequentially filtered using QC parsing with a 30% relative standard deviation (RSD) to screen out features that showed instability or irreproducibility across the dataset, reducing the original feature list. Datasets were grouped by treatment, pre- and post-treatment and RS and NRS groups separately and uploaded to MetaboAnalyst 5.0 [23] for univariate statistical analysis and multivariate chemometric analysis using the statistical analysis (one factor) module. The non-parametric relative standard deviation (median absolute deviation [MAD]/median) was used to filter untargeted metabolomics datasets (i.e., spectral binning data, peak lists) with a large number of variables to screen out baseline noise [24], while log transformation helped to remove heteroscedasticity from the data and correct for a skewed data distribution. Autoscaling was used to remove the dependence of the ranking of the metabolites on the average concentration and the magnitude of the fold changes and showed biologically sensible results after principal component analysis [25]. For biomarker identification, the biomarker analysis module of MetaboAnalyst™ was used, which comprises receiver operating characteristic (ROC) curve analysis and random forest classification models.

## 3. Results

### 3.1. Baseline Characteristics

Forty people received the interventions and 37 people finished the trial. Participants’ characteristics at baseline can be found in Table 1. The average age of the participants was 26 years, with an average BMI of 23 kg/m^2^, and 84% were Caucasian. A food diary was collected to calculate the dietary energy intake throughout the trial, to compare the mean energy and macronutrient intake values in the pre- and post-intervention groups. The homogeneity of variances was tested prior to the analysis (*p* > 0.05). There was no statistical difference in the total energy, carbohydrate, fat and protein intake of participants between and within intervention groups, as we have reported previously [13].

### 3.2. Interventional Effect on Clinical Endpoints

The chemical compounds that constitute whole blueberry are ash, protein, fat, carbohydrates and sugars (glucose, fructose), and the main bioactive components are polyphenols and flavonoids [26]. Freeze-dried blueberry powder has an identical composition but with reduced moisture content. A total polyphenol analysis (TPC) was completed for the blueberry, blueberry powder and placebo capsules using the Folin–Ciocalteu reagent method [27], and the nutrient compositions, including total polyphenol content, were comparable between the freeze-dried blueberry powder and whole blueberry, as reported [13]. Compliance was checked on each study visit using the 1-day food diary. Two participants found out about the placebo during the trial via the questionnaire completed upon the end of the trial, and the 1-day food diary confirmed 92% treatment compliance. The data for these participants were retained for analysis.

As we have recently reported [13], no effect of the interventions was found for SBP, DBP and PWV with covariance adjustment for baseline. Moreover, no effect of the interventions was found for plasma triglycerides (TAG), total cholesterol, HDL-cholesterol, LDL-cholesterol, glucose and nitrite (NO_2_^−^) with covariance adjustment for baseline. Both blueberry supplementation and blueberry powder supplementation led to increased NO_2_^−^ levels (+68.66% and +4.34%, respectively) compared to baseline, whereas the placebo supplementation led to a decrease (−9.10%), although the effect was non-significant. No effect of the interventions on any of the cognitive parameters (serial 3 subtraction and serial 7 subtraction tasks for working memory; immediate and delayed word recall and word recognition for episodic memory; visual analogue scales for calm, alert, content and mental fatigue and vigilance for attention), with covariance adjustment for baseline, were found.

### 3.3. Inter-Individual Variation in Endpoints

**Figure 1:**
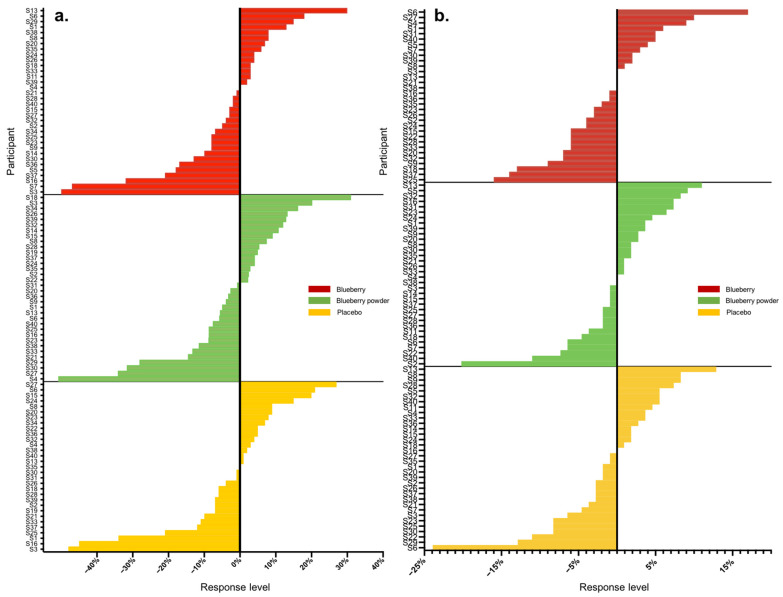
Waterfall plots of individual responses to interventions in endpoints (**a**) PWV, (**b**) SBP.

**Figure 2:**
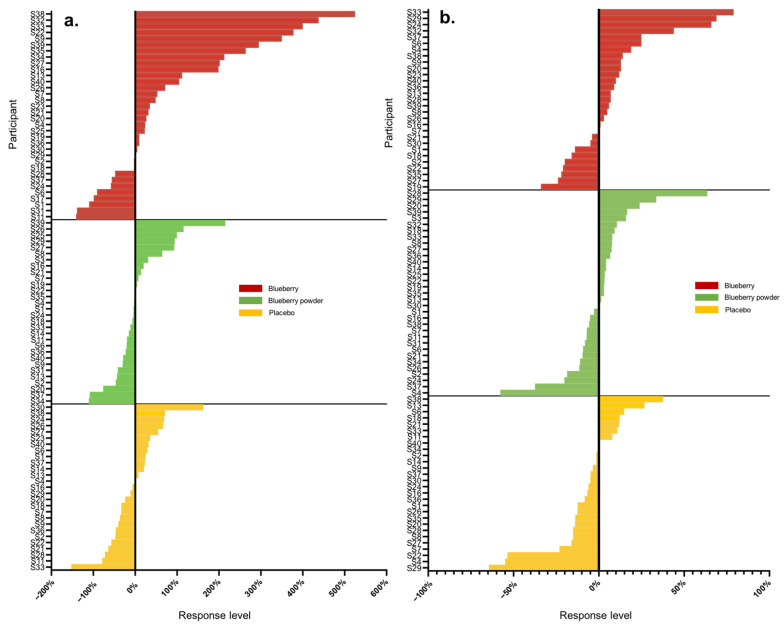
Waterfall plots of individual responses to interventions in endpoints (**a**) nitrite, (**b**) LDL-C.

Overall, we analysed 16 main endpoints, of which seven were associated with cognition and nine were associated with the vascular system. A high range of variation was found for both vascular and cognitive endpoints. Table 2 displays the proportions of participants that showed either improved, worsened or no change in scores compared to the baseline in the assessed endpoints following the blueberry and placebo interventions. In the assessment of vascular function, all participants had changes in their plasma nitrite and lipid levels (triglycerides, total cholesterol), whereas 3–14% of participants did not show changes in the other endpoints. For cognitive function, 3–16% of participants had no changes in most cognitive endpoints except the alertness assessment. The actual level of response was further calculated as a percentage change from baseline, and Table 3 summarises these data for all endpoints with each intervention. Following each blueberry intervention, participants showed dynamic variations for vascular endpoints including nitrite, glucose and lipids, with responses ranging from −141 to +525%. Following the placebo intervention, participants showed a range of responses for vascular endpoints and demonstrated the most dynamic variation for plasma nitrite levels, with −152 to + 163%. The whole-body vascular endpoints, including PWV and blood pressure, presented relatively lower variation, with responses ranging from −51 to +31%. The inter-individual variation in responses for PWV and blood pressure in the placebo intervention group displayed a similar range, between −50 and +30%. A range of moderate to high inter-individual variation was found for cognition, and the most dynamic changes were for self-rated mental fatigue relative to other cognitive endpoints, varying from −114 to +96%. Participants receiving the placebo intervention presented inter-individual variation consistent with that of the blueberry interventions in response to mental fatigue, with a level of −112 to + 89%. The ranges of inter-individual responses are shown as waterfall plots in Figure 1 and Figure 2 for the vascular endpoints.

**Figure 3:**
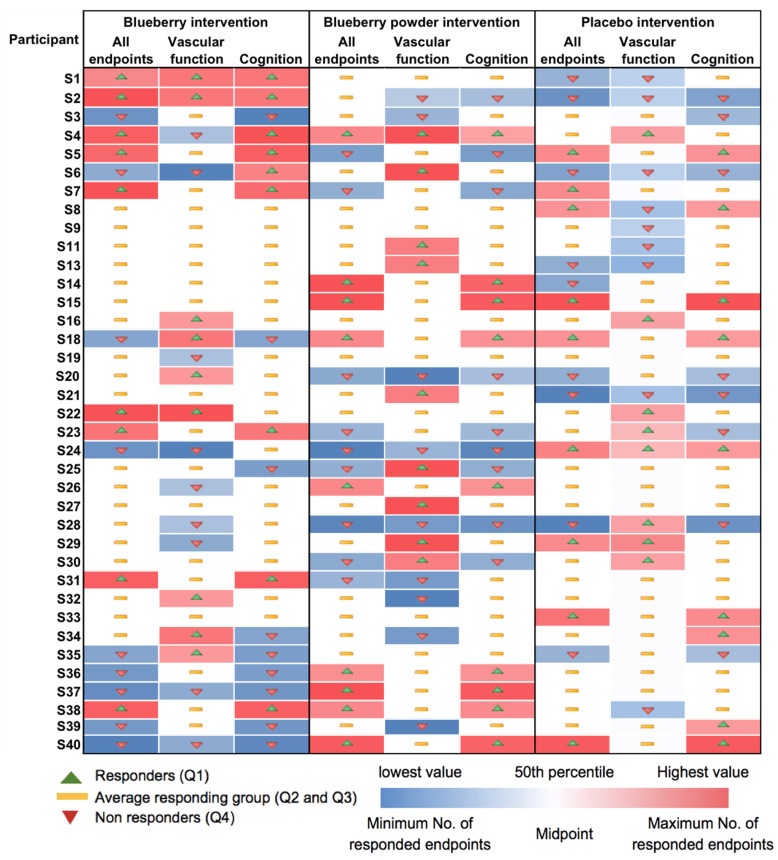
Heat map showing consistency in responses for each participant and for each intervention.

Most participants showed random responses across the vascular and cognitive endpoints following the interventions and also for the placebo control. For example, participant S3 showed the highest response in PWV following the whole blueberry intervention and placebo intervention but had the lowest response for the blueberry powder intervention. For the attention assessment, participant S2 showed the lowest response following the placebo intervention, but moderate and high responses following the blueberry powder and blueberry interventions, respectively. Similarly, participant S18 demonstrated the lowest response following the placebo intervention, but a moderate response following the blueberry powder and blueberry interventions. Participant S2 had improved responses for most vascular and cognitive endpoints following both blueberry interventions. Participant S2 also showed consistently improved responses in the cognitive assessments after the blueberry powder intervention and consistently worsened responses in the cognitive assessments after the placebo intervention, although not in regard to vascular function. Participants S19 and S25 showed consistently improved responses for vascular function following both the blueberry and blueberry powder interventions, but their responses regarding cognitive function appeared random. Figure 2 also highlights the differences between the placebo and treatment groups when there was a larger effect on the endpoint—in this case, plasma nitrite (Figure 2a). The responses for each participant across the endpoints are visualised as a heat map in Figure 3. Again, this figure demonstrates that there was little consistency in the responses across both the vascular and cognitive endpoints in each intervention and even between the similar blueberry treatments.

Participants were classified as normal (18.5–24.9 kg/m^2^) or overweight (25.0–29.9 kg/m^2^), with variations in terms of BMI and gender. An effect of time, in terms of the study visit order, was also found in the intervention for some of the endpoints [13]. Therefore, we tested for an association between gender, BMI and the visit order in the response group using the chi-square test (Table 4). No association between gender and response, BMI and response or visit order and response was found. The participants recruited in the current study were mostly young (97% of participants being less than 42 years old), so the association of age and response to clinical endpoints was not assessed.

### 3.4. Characterisation of Responder and Non-Responder Groups

**Figure 4:**
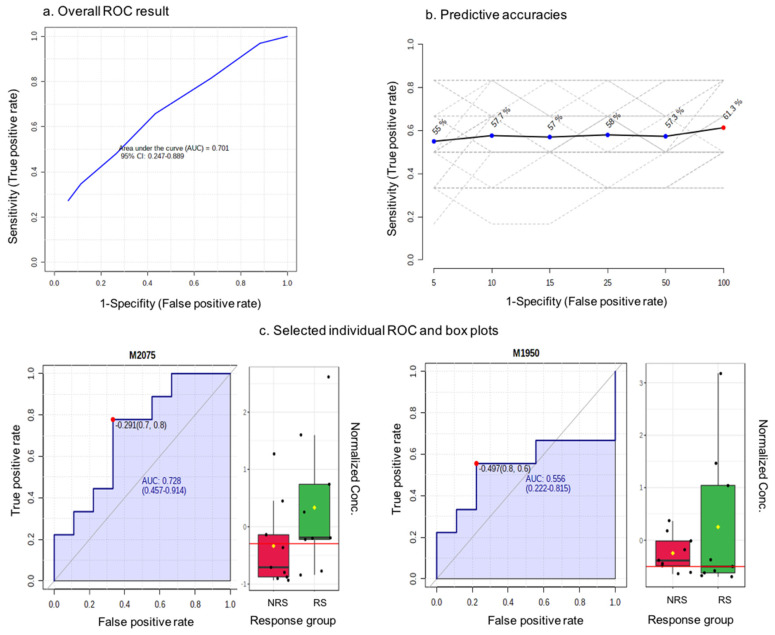
Biomarker identification through receiver operating characteristic (ROC) methodology. red-NRS response group, green-RS response group as presented in x axis; dots corresponding to normalized concentration as shown in y axis.

Only the blueberry powder intervention was used in this exploratory analysis. We used a quartile classification to identify a group of RS and NRS to the intervention based on the number of endpoints that they responded or non-responded to. In total, we identified nine RS and nine NRS, for whom we performed urinary metabolite profiling of the baseline samples, as we wished to identify a predictor of the response. Figure 4a shows the ROC curve for a random forest classification model created using a subset of 100 features selected by the random forest ranking. The area under the curve (AUC) value was 0.701 and the 95% confidence interval (CI) was 0.247–0.889. Meanwhile, the predictive accuracy for the variables was 61.3% (Figure 4b). The predicted class probability for the RS and NRS samples based on the AUC demonstrated that only five out of nine of NS samples and six out of nine NRS samples were predicted correctly. The features were further ranked by their contributions to discriminate between RS and NRS, and 79% of ion features were higher in intensity in NRS samples relative to RS. As any predictive feature should be higher in RS samples, we focused on these ion features, and examples of the area under individual ROC curves and intensity box plots are shown in Figure 4c. The AUCs were modest, ranging from 0.54 to 0.73, and the intensity differences between the NR and NRS samples were not significant. Nevertheless, the most important discriminating species were *m*/*z* 170.069, *m*/*z* 161.094 and *m*/*z* 244.997; however, as they were not significant predictors of the response, their identification was not followed further.

## 4. Discussion

Inter-individual variation needs to be considered when categorising the response to a dietary intervention to help to improve nutrition at the individual level. To our knowledge, this is the first study to characterise the inter-individual variation in a series of clinical endpoints assessing both vascular and cognitive health, and following two very similar dietary interventions with either fresh whole blueberries or blueberry powder. We tested these interventions as it was expected that similar responses to the endpoints would be found. However, we found no consistency in the responses across the interventions or across the various endpoints tested.

### 4.1. Inter-Individual Variation in Responses of Clinical Endpoints

Although we did not find significant treatment effects [13], we did find a large range of responses to the interventions, with 31–71% of participants showing improved responses (up to +11 to +525%) and 29–66% of participants showing worsened responses (up to −12 to −141%) for the vascular and cognitive endpoints. In our meta-analysis on the role of a specific fruit on vascular function, variations (as assessed by SD) for PWV, BP and lipid profiles were reported, ranging from 1.2 to 7.7 and up to 23.6 for plasma nitrite concentrates, following either blueberry powder or juice interventions [9]. Similar to these findings, our current study found the highest inter-individual variation and response in the plasma nitrite levels compared to other vascular assessments. This coincided with the largest treatment effect in plasma nitrite for both blueberry interventions, without the same effect being observed for the placebo intervention; the mean plasma nitrite levels were improved by 68.66% and 4.34% following the whole blueberry and blueberry powder supplementation, respectively, and decreased by 24.5% following the placebo supplementation compared to the baseline. It is likely that the larger the treatment effect, the larger the range of inter-individual responses to an intervention. Plasma nitrite (NO_2_^−^) is converted from nitrate (NO_3_^−^), and the body nitrate (NO_3_^−^) stores can be boosted via the diet (e.g., from leafy vegetables and beetroot) quickly. Approximately 25% of NO_3_^−^ can be absorbed and concentrated in the saliva to be converted to NO_2_^−^ [28]. Therefore, apart from the potential effects following the blueberry interventions, the dynamic conversion of nitrite in the circulating plasma could account for the fluctuations shown for the nitrite responses.

For the endpoints assessing cognition and mood, most participants demonstrated no changes. The cognitive tasks for the current study were selected based on previous evidence of the sensitivity to nutritional interventions [21,29]. The complex regulatory mechanisms of the cerebral blood flow and cognition may explain the lack of intra-individual changes in cognitive performance following the interventions [30]. It is interesting to notice that, following a Mediterranean diet, the inter-individual difference in the alertness score was also higher (+6.93 of response change) compared to contentedness (+5.35 of response change) and calm (−0.28 of response change) based on the visualised rating scale [31]. The current study observed a similar trend for alertness (−70 to +59%) compared to contentedness (−41 to +41%) and calm (−39 to +60%). The inter-individual variations were not limited to the mood assessment though. Inter-individual differences in cerebral blood flow dynamics, neural correlates, heritability, physical and social environment and personality all constitute complex associations between individual responses and specific cognitive domain phenotypes. We have recently shown [10] a larger variation across studies assessing memory (SD of 2.2) relative to executive function (SD of 0.08–0.16) following either a blueberry powder or blueberry concentrate intervention [32,33,34]. However, in the current study, working memory, episodic memory and attention all demonstrated moderate to high variations (−39–+61%), and only one participant showed an improved response across working memory, episodic memory and attention, but not mood, assessments after the blueberry powder intervention. No other participants had a consistent response across the cognitive endpoints, so the response in terms of cognitive abilities also seemed random across domains.

### 4.2. Consistency of Response

We found no consistency in the response across multiple endpoints. Only a very small number of participants (*n* = 2) demonstrated a similar response in the endpoints following both blueberry interventions, and these treatments were chosen to be quite similar in terms of bioactive ingredients; thus, it would be logical to expect some consistency in the endpoint response to them. Consistency in response across the vascular function and cognition endpoints was not expected as they were associated with different biological systems [35], but the endpoints within the same system were expected to show some consistency. For example, it is logical to speculate that similar variations in the BP response and nitrite would be found [36]. However, we observed relatively small variations in SBP (−20 to +17%) and DBP (−34 to +16%) compared to nitrite, and the same participants that responded well to nitrite did not necessarily respond well in terms of BP. Only three participants (out of 37) showed consistent responses within the vascular endpoints; however, there was no consistency in the cognitive endpoints. Most studies report average responses for group effects only, and the variation in response is reported using mostly the SD and coefficient of variation [37,38]. No other study has compared the individual response across cardiovascular and cognitive endpoints.

### 4.3. Predictors of Response

In previous studies, to characterise the response to an intervention, researchers have developed a model or score based on the assessment of health endpoints using a combination of metabolomics, genomics, gut metagenomics, body composition and/or glycaemic responses [38,39,40,41]. A number of interventions have also utilised quartiles, or tertiles, to characterise high or low responses [42,43,44] or used a threshold of improvement (e.g., ≥5% compared to baseline) in a particular clinical endpoint [45]. In the current study, we characterised the response using quartiles and compared the upper (Q1) and lower quartiles (Q4) and NRS groups to identify a predictor of the response using urinary metabolomic profiles.

In the current study, we used the approach of untargeted profiling and ROC analysis to explore the biomarker potential of urinary metabolites in response to vascular and cognitive endpoints. However, 79% of features showed significantly increased intensities in the non-responder group and thus could not be used as biomarkers to predict the response. Several features (e.g., *m*/*z* 170.069, 244.997, 161.094) demonstrated higher intensities in RS relative to NRS but the differences were not significant, and the areas under the curve (0.56–0.72) and specificity (40–60%) did not achieve a high enough level to be predictive of the response to the intervention. Other studies have shown some success in identifying predictors of responses. One randomised controlled trial investigated the metabotype in order to explain the inter-individual variability in the assessment of cardiovascular biomarkers following the use of pomegranate polyphenol extracts [1,46]. The microbial-derived urolithin metabotype UM-B was shown to be solely associated with individual improvements in the response of the blood lipid profile, including total cholesterol (−15.5 ± 3.7%) and LDL-cholesterol (−14.9 ± 2.1%) [46]. Although the individual response was not described, the metabotype could be used to predict responders that could benefit from pomegranate supplementation. In a similar approach, Garcia-Perez et al. developed a prospective dietary metabotype score (DMS) to directly map individual urinary metabolites to differentiate between metabotypes [11]. A ranking was used to further classify individual responses to either a healthy or unhealthy diet based on the WHO guidelines [11]. Their DMS score consisted of nutrient-responsive metabolites including hippurate, 3-methylhistidine and carnitine that were derived from the intake of specific foods. When the metabotypes were profiled following the highly controlled healthy or unhealthy diet, other individual metabotypes in the DMS score could be used to predict the diet that they were adhering to. A significant association between urinary metabotypes and glycaemic and lipid profiles was shown, but the variation was not quantified in their study. Compared to their work, a DMS score could not be applied in the current study as only one unknown feature was found that significantly increased in intensity (*m*/*z* 233.163) following the blueberry intervention, and no metabotype predicting the response was found that would enable the development of a similar ranking scheme. Another recent study (PREDICT trial) reported that a person’s postprandial triglyceride and glucose response to the same meals was often similar and therefore predictable, although the responses were highly variable between individuals [37]. This study measured the inter-individual variation via the coefficient of variation (SD/mean, %) in the population, and, similar to the previous findings, our study observed a small number of RS (2 out of 9) that demonstrated consistent responses in the assessment of the total number of endpoints following both of the blueberry interventions, but the consistency was not shown across vascular and cognitive function. The PREDICT trial recruited a large cohort (*n* = 1002) and measured responses that were all postprandial metabolic endpoints, which could be attributable partially to their consistent findings. It is also worth noting that the study summarised person-specific factors, specifically the gut microbiome, that had a greater influence (7.1% of variance) than did meal macronutrients (3.6%) for some of the postprandial responses, whereas genetic variants had a modest impact on the postprandial predictions. Therefore, to distinguish predictors of individual responses to interventional studies, comprehensive assessments are required.

### 4.4. Methodological Factors Influencing Inter-Individual Variation

Aside from the established sources of inter-individual variation as mentioned in the Introduction, and for which several reviews are available [47,48], certain methodological issues can contribute to the variation. The influence of the visit order (treatment sequence) on the individual response was explored but no association was found. The inter-individual variation in baseline levels was considered as a fixed factor and covariate in the analysis of the intervention effects on the endpoints, and the baseline levels of each endpoint could also influence the individual response following the intervention. Even though the response was presented as a percentage change from the baseline, participants with relatively higher cognitive levels at baseline were unlikely to achieve higher cognitive responses following a dietary intervention due to ‘ceiling effects’ [49]. Similarly, participants with relatively lower levels of vascular dysfunction risks, such as low BP, may be unlikely to achieve a further reduction [50]. Therefore, a larger sample group covering participants with both low and high health risks at baseline may be necessary to investigate the association between baseline health risks and the response in terms of vascular and cognitive endpoints. Apart from the above factors, age, BMI, gender, physical activity, smoking, infection, blood lipids and medication have also been suggested to be responsible for inter-individual differences in the response to a dietary intervention [48]. The current study did not find associations between gender or BMI and response, and the relative importance of each factor in the overall inter-individual differences is still unknown.

### 4.5. Study Limitations

The lack of major effects of the interventions may partially explain the range of responses and limited changes to the urinary metabolome. The metabolomic analysis was also limited to the positive LC-MS mode to provide more features for identification and to check the compliance of participants following the blueberry interventions. The negative ionisation mode and further fragmentation analysis, complemented by the current analysis, would help to confirm the metabolite identifications, as only putative features were identified in this study. Nevertheless, the lack of significant metabolomic changes during the interventions is consistent with the lack of interventional effects shown in the main study [13].

The total polyphenol content, but not the anthocyanin content, in blueberries and freeze-dried blueberry powder was measured and reported in the study [13]. However, the circulating plasma polyphenol concentrations are much lower than the urinary concentrations, whereas the plasma is essential to deliver the polyphenols and their metabolites to the targets and exert their physiological effects; thus, a larger dose may need to be administered to demonstrate efficacy [51]. Although the biokinetics post-intervention in the current study were unknown, the timings of the cognitive effects observed appeared to be closely related to the absorption and metabolism rates of the supplemented fruit. The plasma polyphenols and flavonoids, including quercetin, myricetin and kaempferol and their metabolites, reaching peak intensities at 1–2 h and 6 h following blueberry consumption, have been observed to correspond with two peak timings regarding cognitive effects on memory, executive function and mood [52,53,54]. However, the dosage that the subjects consumed in our study may not have been able to maintain the polyphenol levels within the body after absorption and overnight fasting (>12 h) prior to the study visits, and the acute time–response effect was not assessed in this study. It also should be noted that the batch-to-batch variations in nutritional value and polyphenol content, especially anthocyanins for whole blueberry, were not assessed in the current study [55]. The total polyphenol content for the two interventions was analysed using the Folin–Ciocalteu colorimetric assay, but many chemical compounds may act as interfering agents, such as ascorbic acid in this method, producing inaccurate estimations of the real phenolic compound concentrations in the matrix [56,57].

In the current study, the potential covariances of sex, age, BMI, ethnicity, socioeconomic status and physical activity were not included in the analyses, but we included mostly young participants (25.86 ± 6.81 years old), who were white (*n* = 31) and healthy, with a non-smoking status during the trial. There were significant effects of the visit order observed due to the randomisation of the treatment sequence, and interaction effects between treatments and visits were also observed in the study. A crossover design includes repeated visits and has the limitation of the carryover effect of different treatments, which may explain the influence of the visit order on the treatment effects in the study [58]. There was low retrospective power (<80%) for some of the endpoints; therefore, certain endpoints were underpowered, which could have impacted the ability to detect significant treatment effects for some of the primary endpoints in the current intervention.

Quartile division based on the response levels calculated at the endpoints was utilised to quantify the number of RS and NRS following the interventions. Unfortunately, there was no consistent response identified; thus, the characterisation of the response was not differentiated at the metabolomic level between RS and NRS. This could be partially explained by the relatively small sample size for the RS and NRS groups (9 each) and the inter-individual variation at baseline. Therefore, the predictive features should be explored and validated in a larger population so that a larger number of responses and a wider range of individuals can be classified as RS and NRS. However, it is worth noting that previous therapeutic studies have applied a similar quartile calculation method to characterise the different responses for non-diabetic essential hypertensives following short-term thiazide treatment [59], or for coronary syndrome patients following antiplatelet therapy [60]. Furthermore, genetic mapping for nutrient-responsive and physiologically responsive variants may help to characterise RS and NRS in an intervention based on genotypic feedback.

Currently, very few studies have combined genomics, epigenetics, metabolomics and gut microbiome and anthropometric characteristics together to evaluate individual responses in the assessment of clinical endpoints following dietary interventions. The current study recruited mainly females (71%), and there was no association between gender and response. The above factors could all influence the absorption, distribution, metabolism and excretion (ADME) processes known to influence metabolic phenotypes [48]. Indeed, a range of studies under the COST Action POSITIVe scheme revealed a lack of knowledge about the carriers, enzymes, isoforms and gut bacteria involved in ADME, making it difficult to identify a key biomarker of ADME variability [48]. It is important to understand how much each factor contributes to the inter-individual variation in response (i.e., via linear mixed methods or multivariate analysis) following a dietary intervention in future work.

## 5. Conclusions

We have shown that the response across endpoints following two similar blueberry interventions was inconsistent both between and within participants. No predictive biomarker for the discrimination of responders to the endpoints following the blueberry interventions was identified. Nevertheless, there remain many gaps in our knowledge. More approaches characterising responses in human intervention studies and data coupling with metabolomic, genotypic and lifestyle behaviour feedback will be needed to unravel how they contribute to the inter-individual variation in physiological responses. The findings from the current study suggest that a novel approach characterising the responses across endpoints following a dietary intervention, beyond the ‘one size fits all’ dietary strategy, is necessary prior to defining a beneficial food or food group.

## Figures and Tables

**Table 1:** Baseline demographics and influence of blueberry interventions across multiple endpoints.

Variable	Value ^1^
Age (years)	25.86 ± 6.81
BMI (kg/m^2^)	23.15 ± 3.12
Gender	13 male, 24 female
Ethnicity	1 Black
	2 Indian Asian
	3 Chinese Asian
	31 Caucasian, European
Fruit and vegetable intake (portions/day)	2.13 ± 0.85
Berry intake (portions/day) ^2^	0.06 ± 1.61

^1^ Data are expressed as mean ± SD; BMI: body mass index. ^2^ One portion size equals 80 g.

**Table 2:** Proportions of participants showing different responses in endpoints after blueberry and placebo interventions.

Endpoint	Blueberry Intervention			Blueberry Powder Intervention			Placebo Intervention		
	↑	—	↓	↑	—	↓	↑	—	↓
PWV	49%	6%	46%	53%	0%	47%	56%	3%	41%
SBP	52%	9%	39%	43%	14%	40%	53%	3%	44%
DBP	56%	6%	38%	49%	14%	37%	41%	6%	53%
Nitrite (NO_2_^−^)	71%	0%	29%	37%	0%	63%	48%	0%	52%
Glucose	45%	0%	55%	50%	3%	47%	66%	0%	34%
TAG	34%	0%	66%	59%	0%	41%	34%	0%	66%
Total cholesterol	36%	0%	64%	42%	0%	58%	54%	0%	46%
HDL-C	55%	0%	45%	55%	0%	45%	59%	3%	38%
LDL-C	31%	3%	66%	42%	0%	58%	71%	0%	29%
Working memory ^1^	57%	12%	31%	48%	14%	36%	55%	13%	32%
Episodic memory ^1^	48%	14%	37%	45%	16%	38%	44%	13%	43%
Attention ^1^	47%	11%	42%	50%	8%	42%	45%	3%	52%
Alert ^1^	71%	0%	29%	47%	0%	53%	53%	0%	47%
Content ^1^	54%	11%	35%	33%	6%	61%	59%	0%	41%
Calm ^1^	62%	3%	35%	58%	3%	39%	39%	6%	55%
Mental fatigue ^1^	45%	3%	52%	55%	4%	41%	52%	5%	43%

↑ improved score after intervention; — no change after intervention; ↓ worsened score after intervention; PWV: pulse wave velocity; SBP: systolic blood pressure; DBP: diastolic blood pressure; TAG: triglycerides; HDL-C: high-density lipoprotein cholesterol; LDL-C: low-density lipoprotein cholesterol. ^1^ Multiple task parameters (43) were used to assess the 7 cognitive domains tested, so a combined mean value of the proportions of participants that responded to these multiple tasks under the assessed cognitive domains was calculated instead.

**Table 3:** Range of responses (%) in endpoints after blueberry and placebo interventions.

Endpoint *	Response (%)		
	Blueberry Intervention	Blueberry Powder Intervention	Placebo Intervention
PWV	−48–+27%	−51–+31%	−50–+30%
SBP	−16–+17%	−20–+11%	−24–+13%
DBP	−34–+16%	−28–+12%	−33–+25%
Nitrite (NO_2_^−^)	−141–+525%	−111–+215%	−152–+163%
Glucose	−33–+66%	−32–+25%	−33–+44%
TAG	−105–+95%	−94–+132%	−97–+132%
Total cholesterol	−30–+62%	−68–+43%	−45–+32%
HDL-C	−51–+85%	−90–+52%	−35–+26%
LDL-C	−34–+79%	−58–+64%	−64–+38%
Working memory	−39–+61%	−21–+51%	−35–+55%
Episodic memory	−30–+30%	−12–+21%	−20–+24%
Attention	−33–+18%	−34–+14%	−19–+19%
Alert	−57–+48%	−70–+59%	−40–+39%
Content	−41–+42%	−33–+28%	−27–+26%
Calm	−39–+60%	−29–+40%	−109–+24%
Mental fatigue	−65–+96%	−114–+80%	−112–+89%

* PWV: pulse wave velocity; SBP: systolic blood pressure; DBP: diastolic blood pressure; TAG: triglycerides; HDL-C: high-density lipoprotein cholesterol; LDL-C: low-density lipoprotein cholesterol.

**Table 4:** Association between gender, BMI and study visit order with inter-individual response.

Chi-Square Test Statistics ^1^					
	Factor	Response Following Blueberry Intervention		Response Following Blueberry Powder Intervention	
All endpoints	Gender	X^2^ = 1.000	*p* = 0.620	X^2^ = 1.286	*p* = 0.576
	BMI	X^2^ = 0.000	*p* = 1.000	X^2^ = 0.000	*p* = 1.000
	Visit	X^2^ = 0.000	*p* = 1.000	X^2^ = 1.067	*p* = 0.587
Vascular Function	Gender	X^2^ = 0.234	*p* = 1.000	X^2^ = 1.000	*p* = 0.620
	BMI	X^2^ = 0.234	*p* = 1.000	X^2^ = 0.000	*p* = 1.000
	Visit	X^2^ = 0.533	*p* = 0.766	X^2^ = 0.900	*p* = 0.638
Cognition	Gender	X^2^ = 1.000	*p* = 0.620	X^2^ = 3.600	*p* = 0.206
	BMI	X^2^ = 0.234	*p* = 1.000	X^2^ = 0.000	*p* = 1.000
	Visit	X^2^ = 1.111	*p* = 0.574	X^2^ = 1.067	*p* = 0.587

^1^ Degree of freedom = 1; sample size (N) = 18; 2-sided alpha level of 0.05 significance.

## Data Availability

The data presented in this study are available on request from the corresponding author.

## Supplementary Materials

The following supporting information can be downloaded at: https://www.mdpi.com/article/10.3390/nu16060895/s1. A list of foods to avoid during the dietary interventions was provided.
