# Supplementary material for: Inter-Individual Responses to a Blueberry Intervention across Multiple Endpoints
Source: Nutrients. 2024 Mar 20;16(6):895. doi: 10.3390/nu16060895 (PMC10975049; doi:10.3390/nu16060895)
Supplement: Supplementary file 1 [file nutrients-16-00895-s001.zip › nutrients-2881199-supplementary.pdf]

Note to participants:

Kindly please note that following listed “purple foods” should be avoided during your participation in this study.

**Berries**
1. Blueberries<br>2. Blackberries<br>3. Blackcurrant<br>4. Concord grapes (dark/purple)<br>5. Cherries<br>6. Chokeberries<br>7. Raspberries<br>8. Strawberries
**Other fruits**
1. Plums<br>2. Prunes<br>3. Raisins
**Veggies**
1. Beetroot<br>2. Aubergine<br>3. Red cabbages<br>4. Red onions
**Juices**
All smoothie/juices blended from above foods
**Alcohol**
1. Red wine
**Other foods**
1. Pastry, cake or biscuits containing cherries and berries
2. Cherries/berries containing muesli and fruit flakes
